# Supplementary material for: Depot-specific mRNA expression programs in human adipocytes suggest physiological specialization via distinct developmental programs
Source: PLoS One. 2024 Oct 14;19(10):e0311751. doi: 10.1371/journal.pone.0311751 (PMC11472956; doi:10.1371/journal.pone.0311751)
Supplement: S1 Table — List of adipocyte sample information including adipose depot of origin, patient gender and age, date of collection/adipocyte isolation, and any other known information such as surgical procedure. F = female, M = male, followed by the age of the patient; U denotes either unknown patient sex or age. Lowercase letters are used to distinguish patients with the same sex and age. In the notes column “not OW/obese” indicates that the patient was not overweight or obese. (DOCX) [file pone.0311751.s001.docx]

###
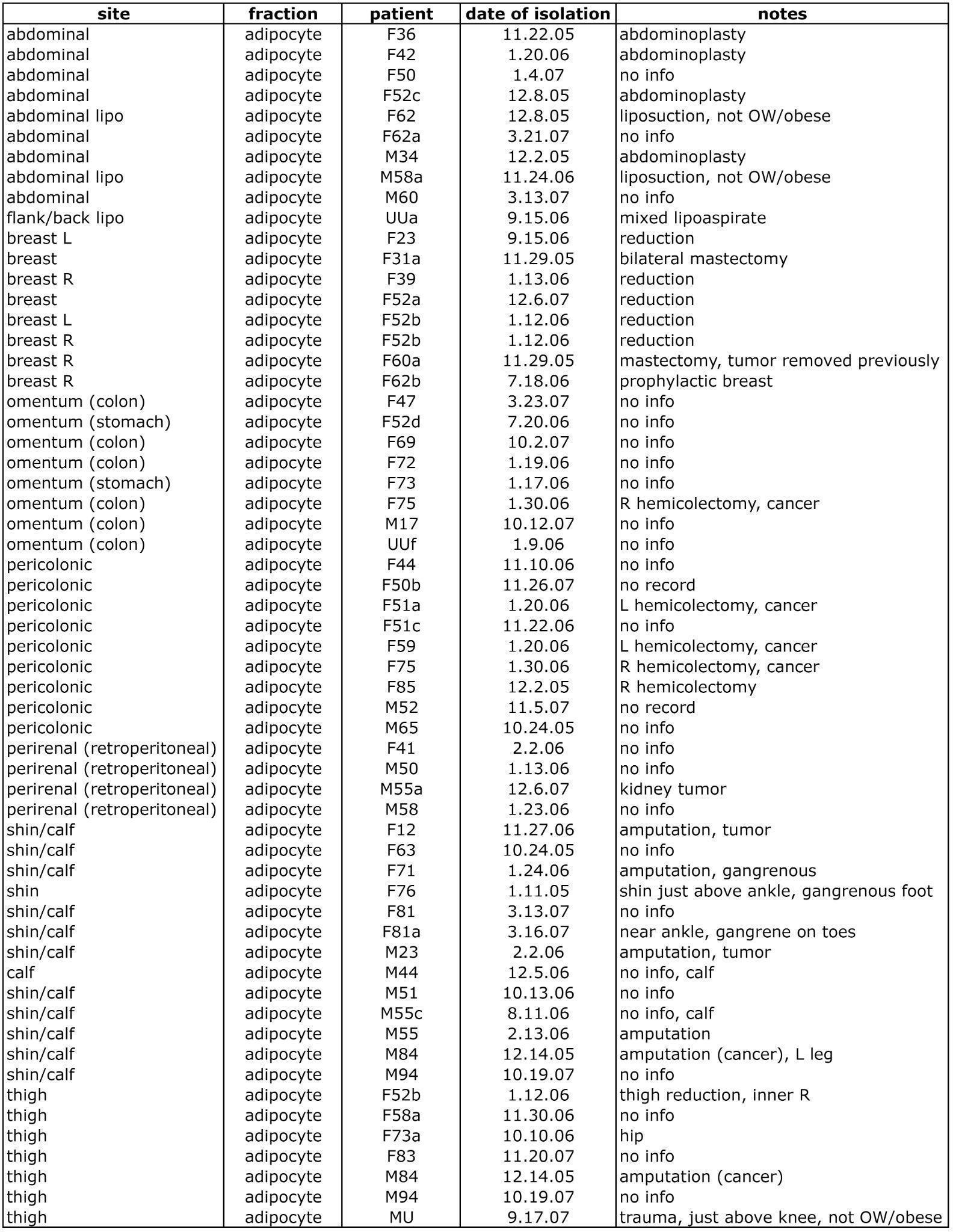


**S1 Table. Detailed information for adipocyte samples collected.**

List of adipocyte sample information including adipose depot of origin, patient gender and age, date of collection/adipocyte isolation, and any other known information such as surgical procedure. F = female, M = male, followed by the age of the patient; U denotes either unknown patient sex or age. Lowercase letters are used to distinguish patients with the same sex and age. In the notes column “not OW/obese” indicates that the patient was not overweight or obese.
