# Supplementary material for: Depot-specific mRNA expression programs in human adipocytes suggest physiological specialization via distinct developmental programs
Source: PLoS One. 2024 Oct 14;19(10):e0311751. doi: 10.1371/journal.pone.0311751 (PMC11472956; doi:10.1371/journal.pone.0311751)
Supplement: S2 Table — For each gene shown in S7–S10 Figs, the table lists the Pearson correlation and associated p-value between that gene’s relative expression measured by microarray vs. RT-qPCR, using the 46 samples profiled on both platforms. (DOCX) [file pone.0311751.s002.docx]

| Figure | gene | Pearson r | p-value |
| --- | --- | --- | --- |
| S7 | TBX15 | 0.96 | 4E-25 |
| S7 | SOX9 | 0.81 | 9E-12 |
| S7 | SHOX2 | 0.88 | 3E-15 |
| S7 | HOXC8 | 0.95 | 1E-24 |
| S7 | HOXA13 | 0.84 | 5E-13 |
| S7 | HOXB7 | 0.53 | 0.0001 |
| S7 | NKX3-2 | 0.54 | 0.0008 |
| S7 | FOXD1 | 0.51 | 4E-04 |
| S7 | IRX1 | 0.02 | 0.9 |
| S7 | TWIST1 | 0.97 | 2E-27 |
| S7 | ZIC1 | 0.81 | 3E-09 |
| S7 | HOXB8 | 0.66 | 1E-06 |
| S8 | PPARG1 | 0.71 | 3E-08 |
| S8 | ACACA | 0.88 | 1E-15 |
| S8 | ACACB | 0.79 | 6E-11 |
| S8 | FASN | 0.93 | 7E-21 |
| S8 | PFKFB3 | 0.34 | 0.02 |
| S8 | GYS1 | 0.84 | 3E-13 |
| S9 | PPARG2 | 0.35 | 0.02 |
| S9 | LIPE | 0.36 | 0.01 |
| S9 | CIDEC | 0.09 | 0.55 |
| S9 | PLIN1 | 0.47 | 0.001 |
| S9 | GPD1 | 0.34 | 0.02 |
| S9 | CD36 | 0.23 | 0.1 |
| S9 | LPL | 0.53 | 0.0001 |
| S9 | AQP7 | 0.76 | 1E-09 |
| S10 | ADIPOQ | 0.54 | 1E-04 |
| S10 | LEP | 0.31 | 0.04 |

**S2 Table. Concordance between microarray and RT-qPCR results.**

For each gene shown in SFigures 7-10, the table lists the Pearson correlation and associated p-value between that gene's relative expression measured by microarray vs. RT-qPCR, using the 46 samples profiled on both platforms.
