# Supplementary figures and images for: Depot-specific mRNA expression programs in human adipocytes suggest physiological specialization via distinct developmental programs
Source: PLoS One. 2024 Oct 14;19(10):e0311751. doi: 10.1371/journal.pone.0311751 (PMC11472956; doi:10.1371/journal.pone.0311751)

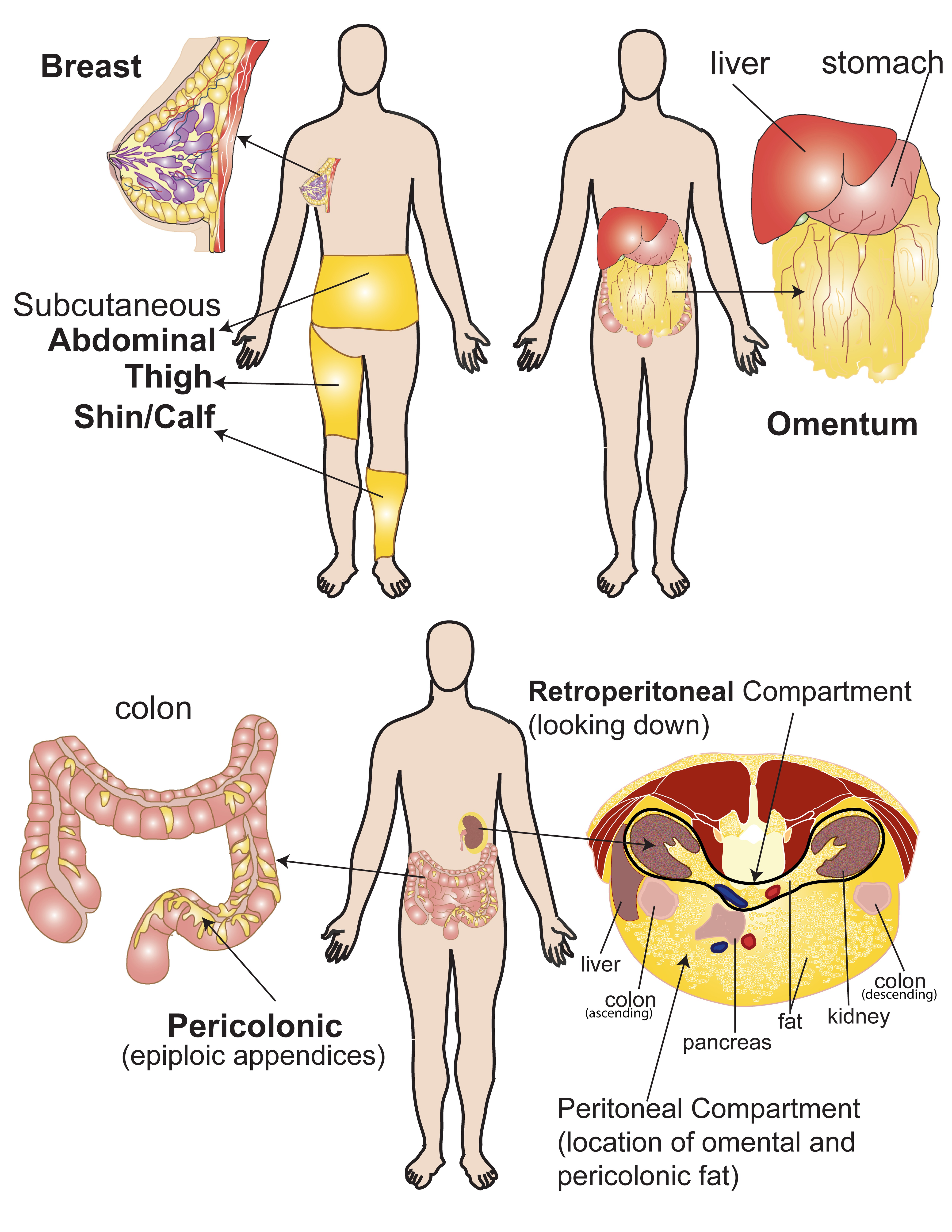

Supplement: S1 Fig — Subcutaneous depots were defined as fat found just underneath the skin: abdominal subcutaneous ranging from anywhere underneath the skin on the ventral side of the body between the rib cage and pelvis, thigh ranging anywhere from the hip to 2 inches above the knee spanning the entire circumference of the leg, shin/calf anywhere below the knee but above the ankle spanning the circumference of the leg. Breast fat samples were either directly subcutaneous, interdigitated with the mammary glands, or a combination of these. Omental and pericolonic depots are both located within the peritoneal cavity (see cross section). Omental fat is a component of the omentum, consisting of a mesothelial pouch that encloses adipose tissue embedded in loose connective tissue and interspersed with aggregates of phagocytic cells termed “milky spots”. The omentum hangs like an apron from the stomach, covering the abdominal organs connecting with the transverse colon and posterior abdominal wall. Pericolonic or epiploic appendices are small pouches of peritoneum filled with fat and situated along the outer surface of the colon and upper part of the rectum. Retroperitoneal fat was excised from the retroperitoneal space, the compartment behind the peritoneum that houses the kidneys, bladder and adrenal glands (see cross section); the surgical samples were labeled “perinephric”, “perirenal”, or simply “retroperitoneal”. (TIF) [file pone.0311751.s003.tif]

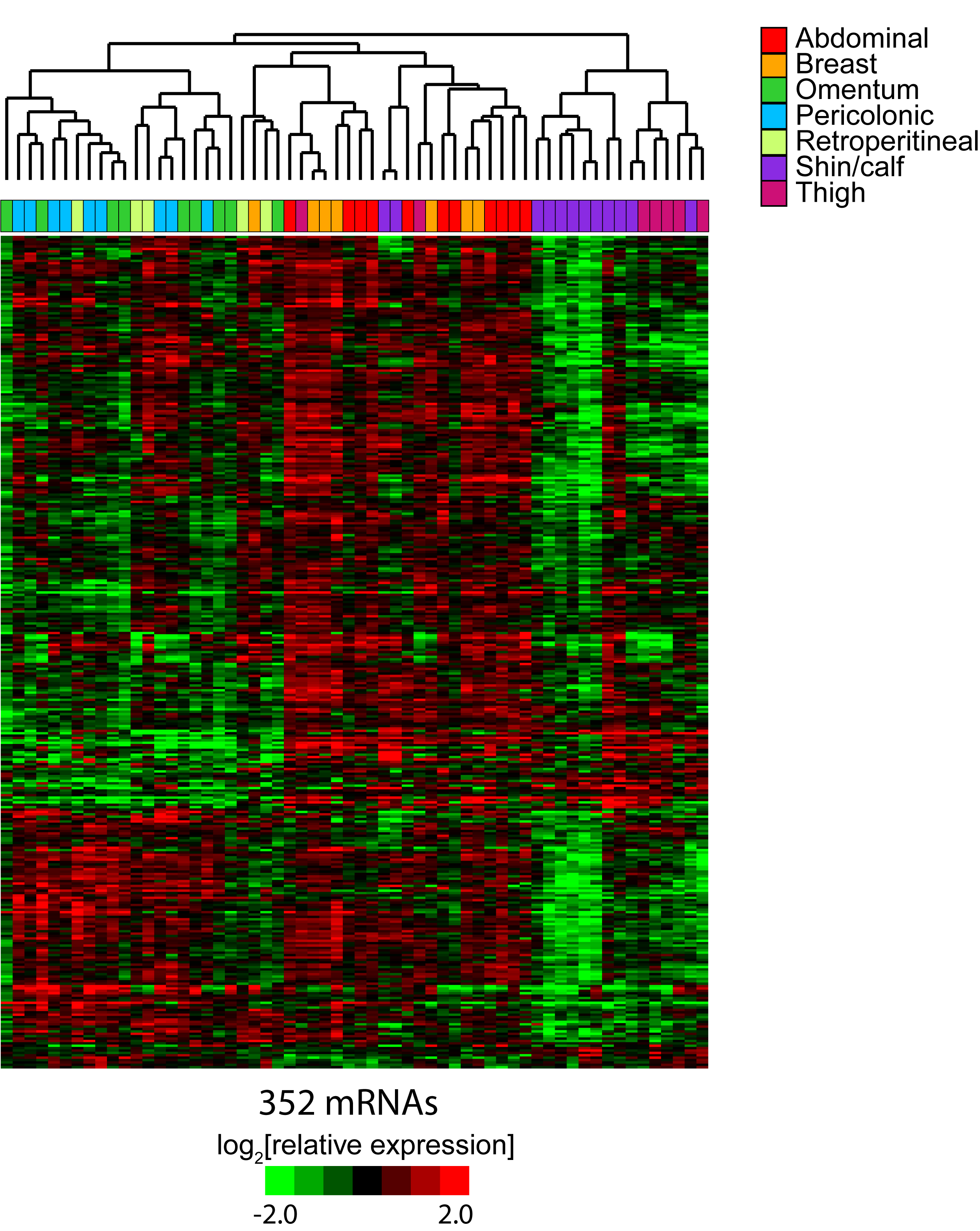

Supplement: S2 Fig — Supervised hierarchical clustering of mRNAs whose variation in expression is largely explained by inter-depot differences (multi-class SAM FDR < 0.2%) and whose expression in adipocytes or ASCs was affirmed by single cell RNA sequencing [58]. (TIF) [file pone.0311751.s004.tif]

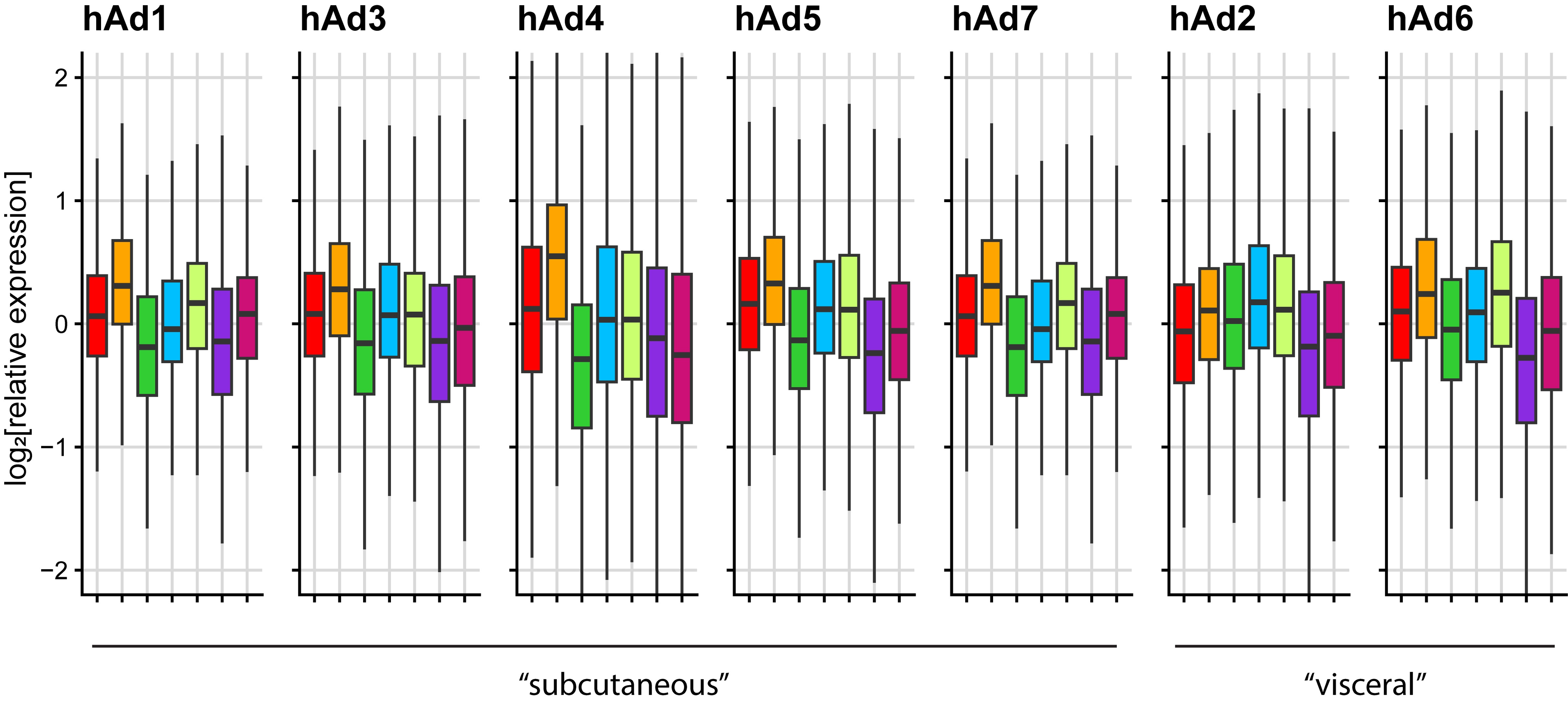

Supplement: S3 Fig — Boxplot representations of the relative expression across depots for seven gene sets that mark distinct adipocyte populations according to [58]. hAD1,3,4,5 and 7 were identified from abdominal subcutaneous adipocytes and hAD2 and 6 were identified from omentum and peritoneal visceral adipocytes. (TIF) [file pone.0311751.s005.tif]

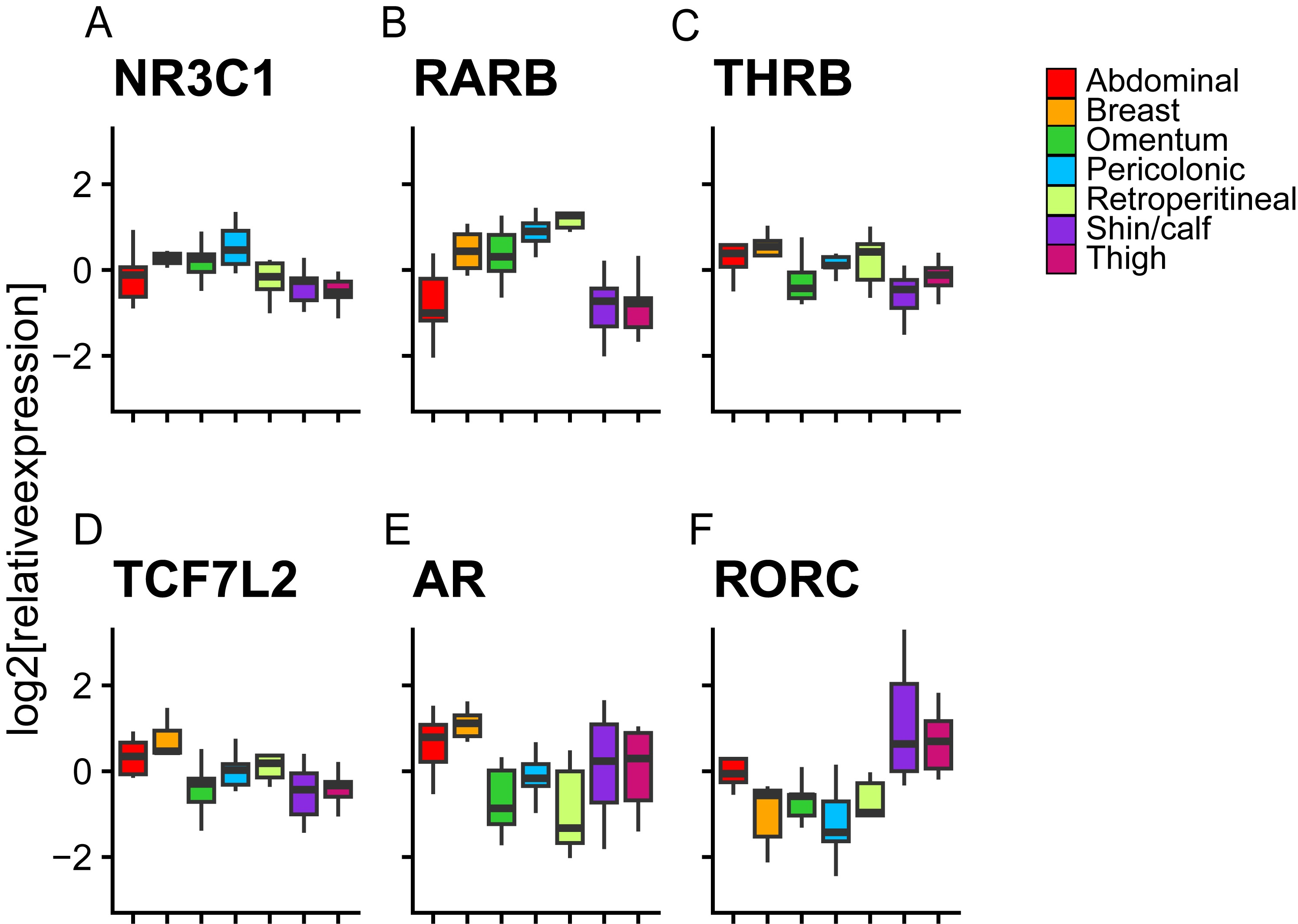

Supplement: S4 Fig — (A-F) Boxplot representation of the relative expression of the indicated gene across depots. (TIF) [file pone.0311751.s006.tif]

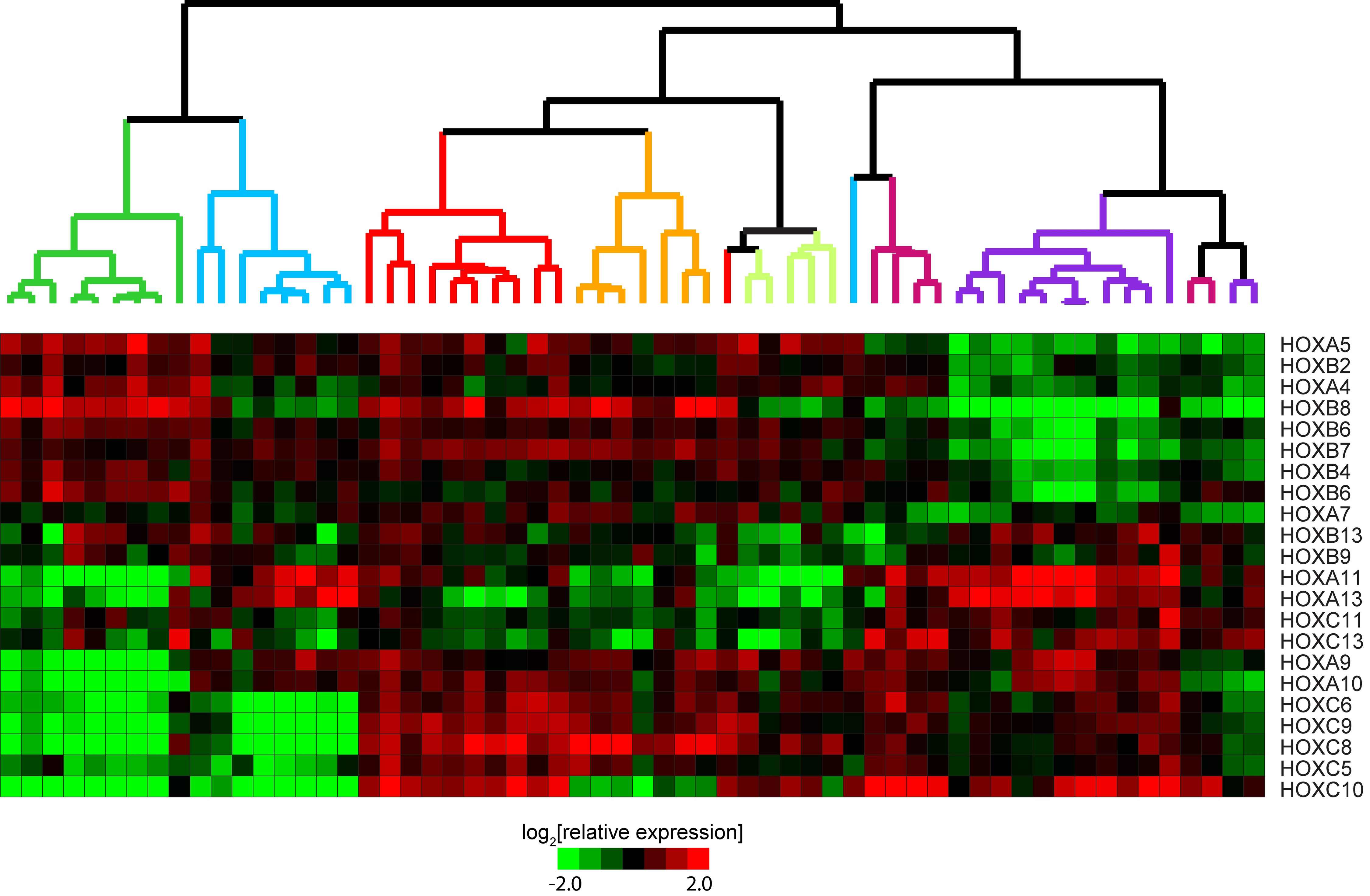

Supplement: S5 Fig — Supervised hierarchical clustering of HOX genes across adipocytes. (TIF) [file pone.0311751.s007.tif]

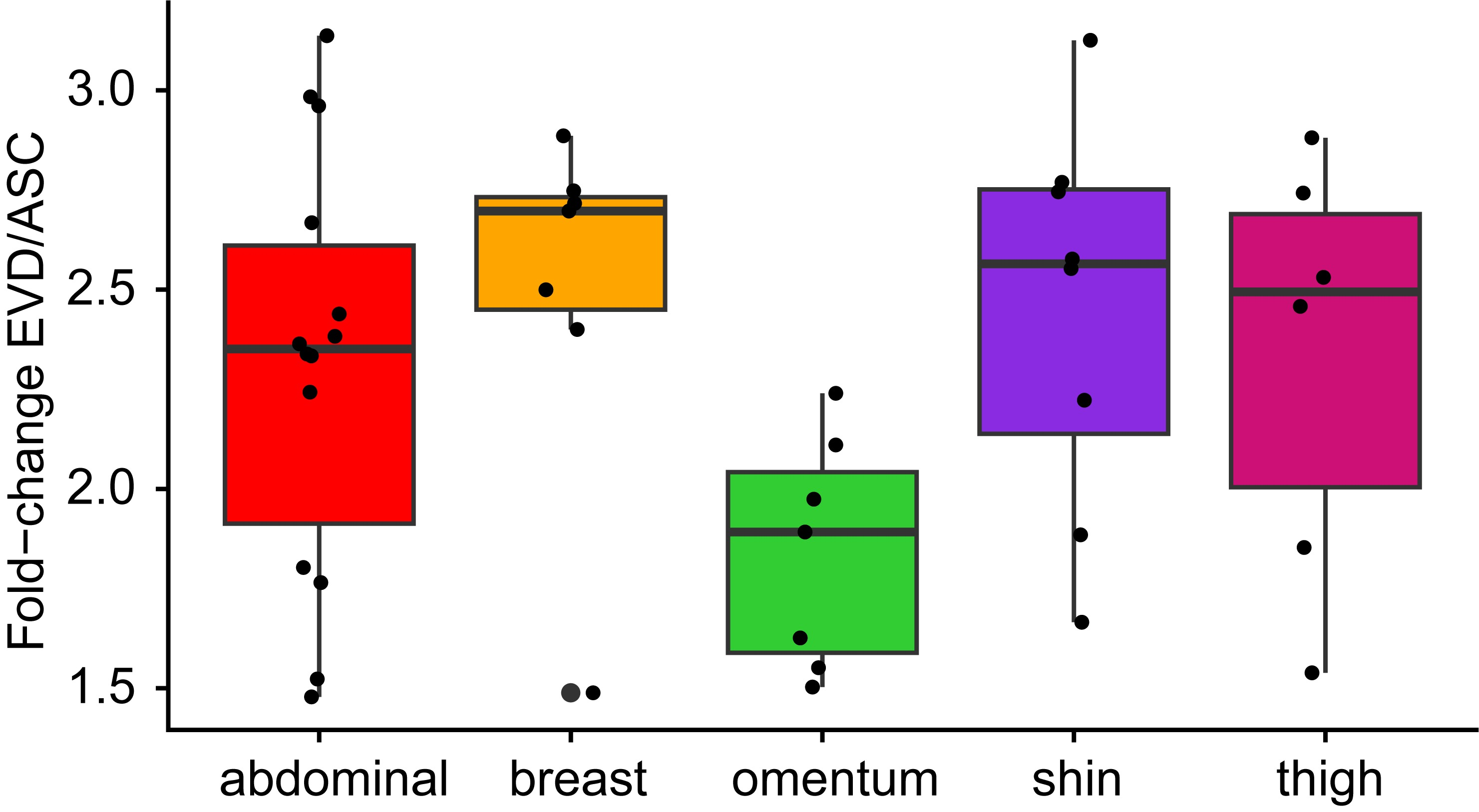

Supplement: S6 Fig — Boxplots of the relative fold-change of PPARγ targets in EVD cells vs. ASCs. Each point represents the average fold-change of the PPARγ targets from one sample. To calculate the fold-change for each mRNA following differentiation, the average log2 expression value across ASC samples from that site was subtracted from the log2 EVD adipocyte expression value. (TIF) [file pone.0311751.s008.tif]

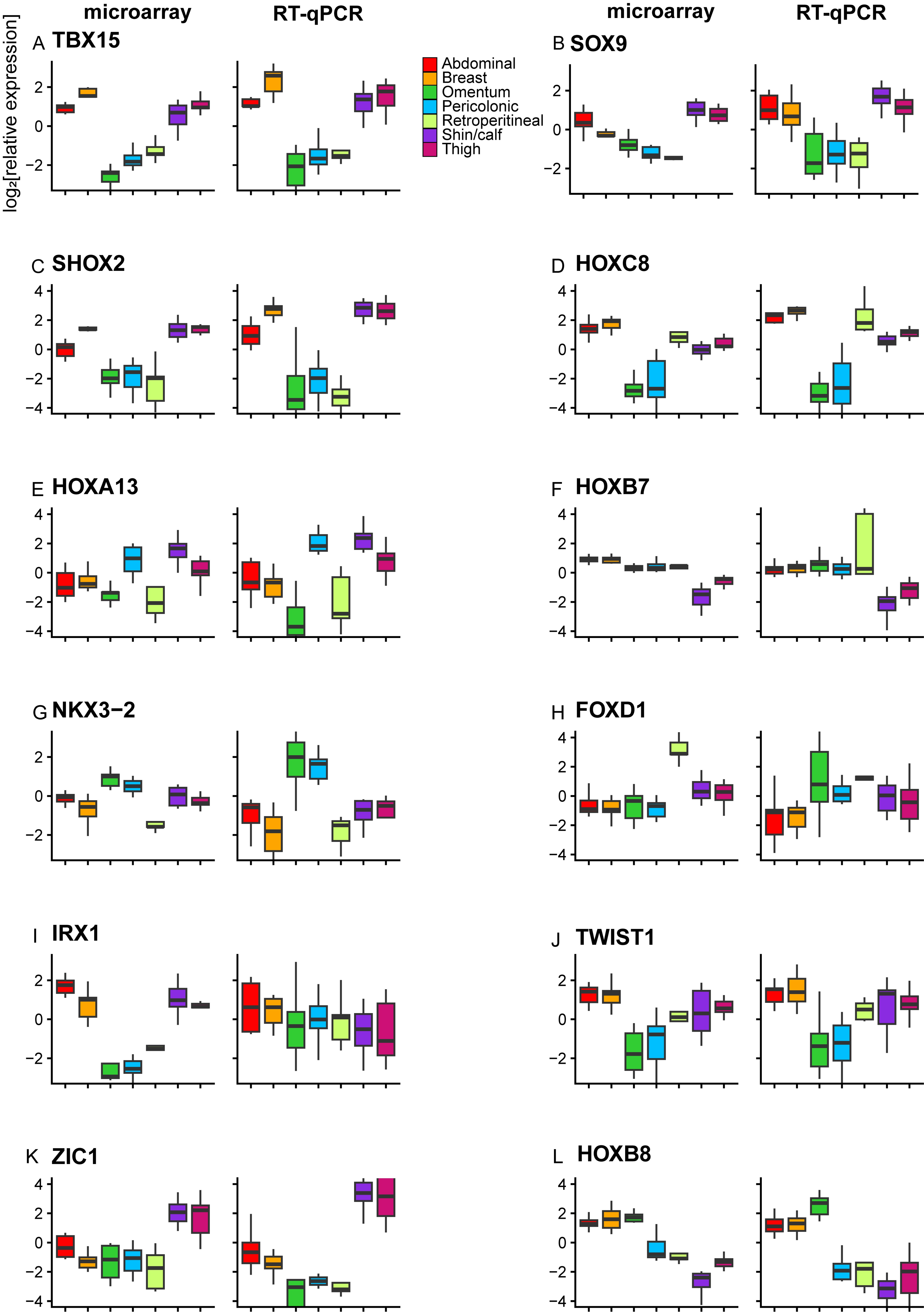

Supplement: S7 Fig — (A-L) Boxplots of the relative expression of the indicated gene in each site according to microarray (left) and RT-qPCR (right). (TIF) [file pone.0311751.s009.tif]

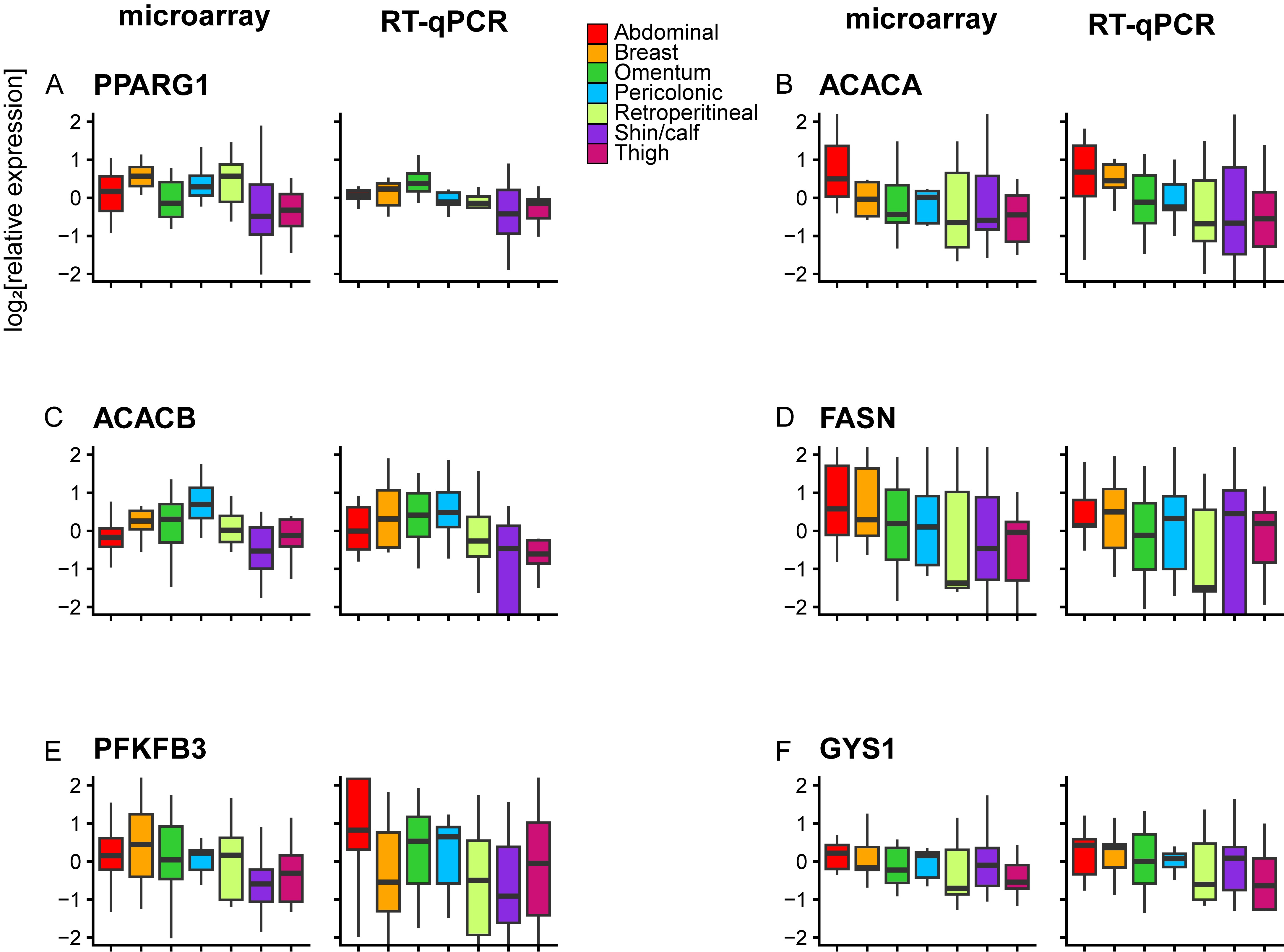

Supplement: S8 Fig — (A-F) Boxplots of the relative expression of the indicated gene in each site according to microarray (left) and RT-qPCR (right). (TIF) [file pone.0311751.s010.tif]

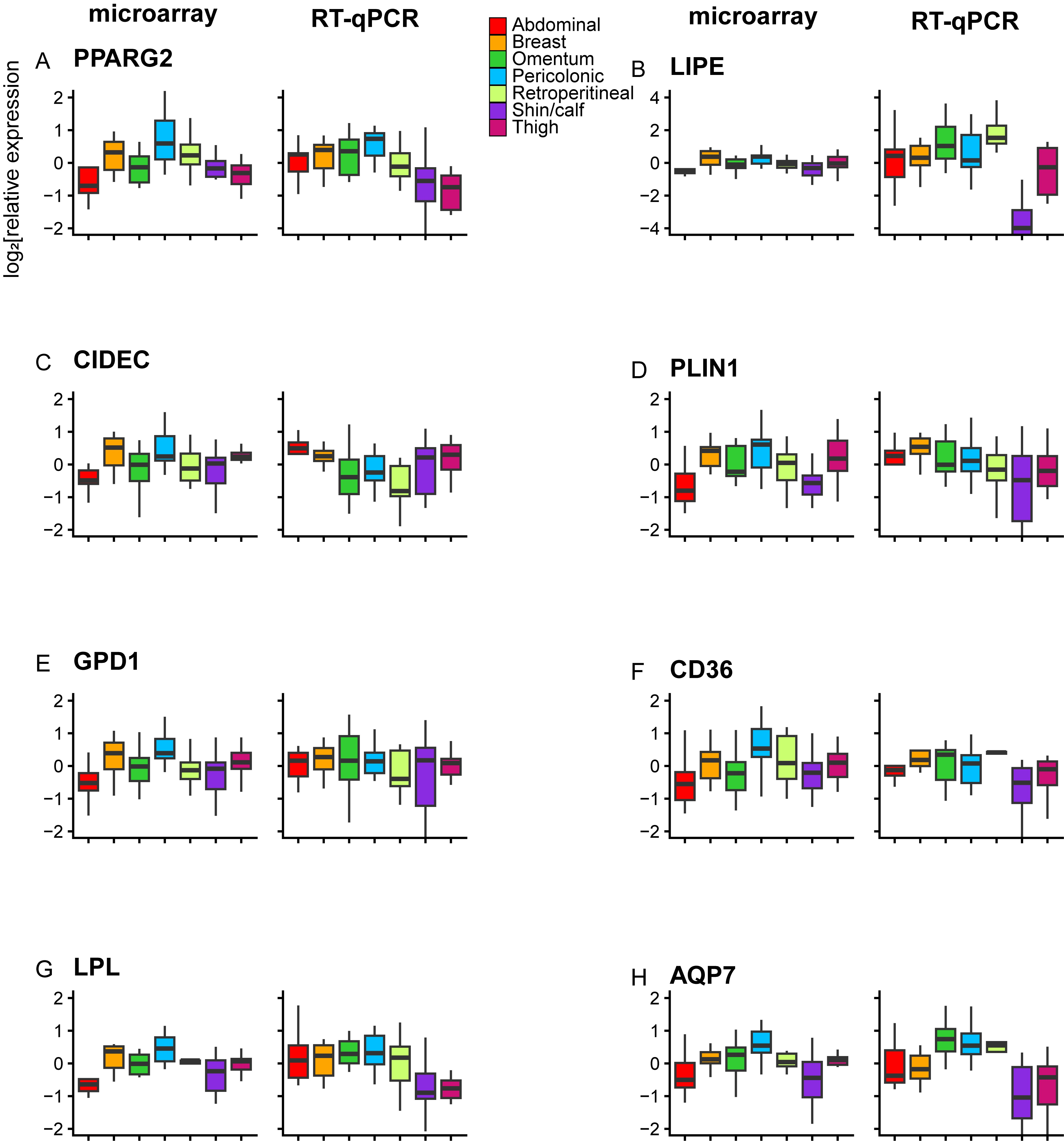

Supplement: S9 Fig — (A-L) Boxplots of the relative expression of the indicated gene in each site according to microarray (left) and RT-qPCR (right). (TIF) [file pone.0311751.s011.tif]

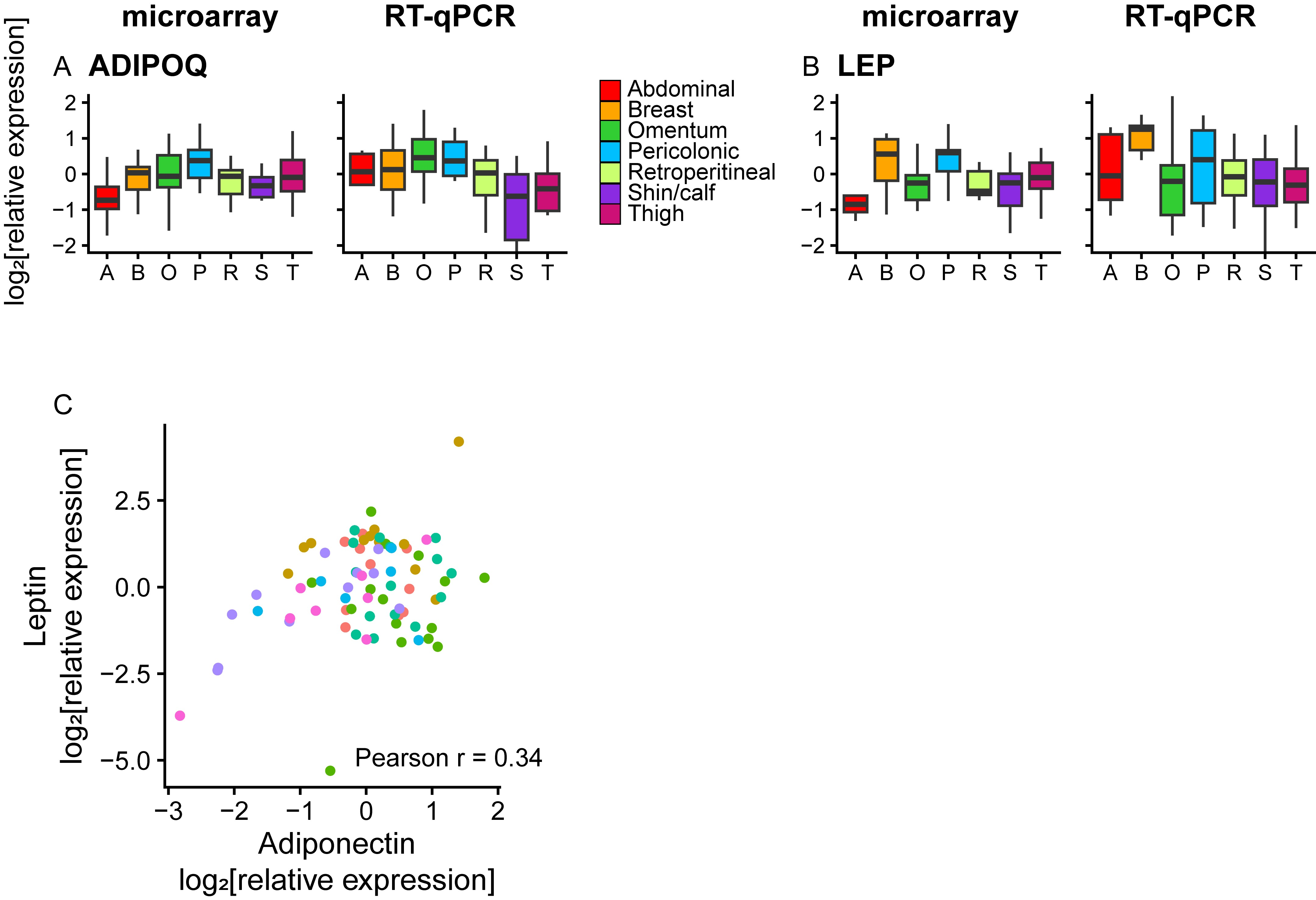

Supplement: S10 Fig — (A) Boxplots of the relative expression of ADIPOQ in each site according to microarray (left) and RT-qPCR (right). (B) Boxplots of the relative expression of LEP in each site according to microarray (left) and RT-qPCR (right). (C) Scatterplot comparing the relative expression of adiponectin (ADIPOQ) and leptin (LEP) across samples, as measured by RT-qPCR. Sample depot is color-coded. (TIF) [file pone.0311751.s012.tif]
