# Supplementary material for: Depot-specific mRNA expression programs in human adipocytes suggest physiological specialization via distinct developmental programs
Source: PLoS One. 2024 Oct 14;19(10):e0311751. doi: 10.1371/journal.pone.0311751 (PMC11472956; doi:10.1371/journal.pone.0311751)
Supplement: Attachment — Submitted filename: Hogan etal 2024.docx [file pone.0311751.s024.docx]

**General Critique:** This is a well-written manuscript that assesses differential gene expression programs between not only visceral versus subcutaneous fat but various anatomic locations, including omentum, pericolonic, retroperitoneal, and abdominal, breast, shin, and thigh subcutaneous areas. The premise of the work is that the location of adipose tissue will be inherently programmed during fetal life and will make the tissue gene expression suite similar to the surrounding tissues (ie, skeletal muscle, liver etc) but dissimilar to the gene expression suite of adipose tissue located in other areas. This premise is supported by the similarity between gene expression in the stromal vascular fraction (SVF) and induced adipocytes in cell culture. This paper is exceptionally comprehensive, and the data analysis overall is appropriate and thorough. However, one very significant issue with the paper is the structure in that much discussion and references about individual genes or findings occur in the results rather than the discussion section. This makes the discussion somewhat vague and less detailed and also breaks up the results section. This reviewer suggests that all discussion and references be moved into the discussion section and be reworked for the flow of the manuscript. Additionally, the authors switch between present and past tense in the discussion of the literature in the introduction. This reviewer suggests using present tense (ie, “X study finds abcd”). The abstract is also very vague as to the main findings of the manuscript. The abstract should be reworked to mention the main driving points of the manuscript more specifically. One drawback to the data analysis and the discussion also is the pooling of data from both sexes. It is well-known that sex (i.e., XX vs. XY) and sex steroids play a role in the propensity for adipose tissue deposition in specific subcutaneous adipose tissue locations as well as viscerally. This is completely ignored in the data set. The authors do have the sex and age of the patient samples. It is the opinion of this reviewer that the authors need to address sex*location interaction in the data set up front first thing. If they can demonstrate no significant interaction between these two variables and share that data as a supplemental figure or table then they can proceed with the pooled-sex analysis as they have already done. Specific critiques are as follows:

**Specific Critiques:**

1) **Results**: Although the manuscript line numbers are not indicated, under the Results section entitled “Maintenance of site-specific differences ex vivo” there is a section of text that has already been mentioned in the M&M. The authors should remove this text from the Results section.
2) **Discussion**: don’t refer back to figures specifically in the discussion
3) **Figures**:
   a. Figure 2: the section under (A) is already mentioned verbatim in the text. Pick a spot where you want this text and put it either in the results text OR in the figure legend. Not both. The figure legend text for B-F is redundant; reword so that you are not saying “same as (B) except for…” for each panel.
   b. Figure 5: Streamline the figure legend text for B-J so it is not redundant (see comment for Figure 2 above). Although it may be somewhat self-evident, define the abbreviations in panels B-J (ie, A, B, O etc) in the figure legend.
   c. Figure 6 B and C: this reviewer is really underwhelmed by the presentation of data in panels B and C here. I feel like there is a more impactful way to show the correlation of the top 200 mRNAs. Please explain why you chose this approach or rework the data to show panels in this figure that are more impactful. Ultimately, the omentum has the worst adipocyte induction in culture, so there is the least gene variation between the 3 sampling conditions. The shin appears to vary more so.
