# Supplementary material for: Depot-specific mRNA expression programs in human adipocytes suggest physiological specialization via distinct developmental programs
Source: PLoS One. 2024 Oct 14;19(10):e0311751. doi: 10.1371/journal.pone.0311751 (PMC11472956; doi:10.1371/journal.pone.0311751)
Supplement: Attachment — Submitted filename: Response to Reviewer PONE-D-24-04146R2.pdf.docx [file pone.0311751.s026.docx]

The only request was from Reviewer #3: *In Figures 1C, 5B-J, and 7B-C, it is crucial to show the statistical analysis results (significance) for the relative gene expression between different depots. This should be highlighted both in the figures and the text description.
Please also indicate the significance for the RT-qPCR data presented in Figures S7, S8, S9, and S10 (A, B).*

We added FDRs for Figure 1C. We already reported in the figure legend and in the main text that the FDRs for all mRNAs in Figure 5 are < 0.5%.

It is not clear what the reviewer means by “significance” for the RT-qPCR data in Figures S7-10. So we guessed that the reviewer wants the significance of the correlation between the array and qPCR results. We added a supplemental table (Table S2) with the Pearson correlation for each gene and the associated p-value.

For Figure 7B and 7C, we respectfully decline to add “significance”. P-values, especially for Figure 7C would be misleading – the fold changes are tiny (compared to 7B), but because we are comparing hundreds of samples and tens of genes, it is easy to get a “significant” p-value – the magnitude of the differences in gene expression are what matters and they are shown in the figures.
